# Therapeutic Metaphors Enhance Memory Systems in Mental Health Contexts

**DOI:** 10.1002/brb3.70270

**Published:** 2025-01-19

**Authors:** Fei Yu, Zhijie Zhang, Wencai Zhang

**Affiliations:** ^1^ Department of Psychology Hebei Normal University Shijiazhuang the People's Republic of China; ^2^ Key Laboratory of Mental Health Institute of Psychology Chinese Academy of Sciences (CAS) Beijing the People's Republic of China

**Keywords:** declarative memory, insight, medial temporal gyrus, therapeutic metaphor

## Abstract

**Background:**

Psychotherapeutic memory plays an important role in maintaining therapeutic effects; however, the neural mechanisms of therapeutic metaphor promoting long‐term memory were still unknown.

**Objective:**

This study used metaphorical micro‐counseling dialog scenarios to investigate the memory effect of therapeutic metaphor and correlated neural mechanisms.

**Methods:**

At first, 31 participants read a mental distress problem, followed by a metaphorical or a literal solution, while undergoing functional magnetic resonance imaging scanning during the encoding phase. One week later, a recognition memory test was performed outside the scanner.

**Results:**

The results revealed that metaphorical solutions were associated with higher insight experiences and better memory performance than literal solutions. Greater activations were observed in the multiple memory systems, including episodic (parahippocampal gyrus, hippocampus, and thalamus), emotional (amygdala), and procedural/implicit (caudate, putamen, and cerebellum), in contrast to later remembered versus later forgotten based on the gap between metaphorical and literal solutions. Insightfulness and activities of the hippocampus, caudate, and cerebellum could predict memory performance.

**Conclusions:**

These findings indicated that multiple memory systems are involved in successful memory encoding of therapeutic metaphors; this suggested that incorporating metaphors into psychotherapy practices could lead to better retention of therapeutic information and improve clinical outcomes compared to literal psychotherapy.

## Graphical abstract

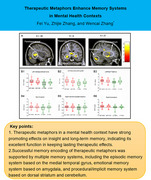

## 1 Introduction

Psychotherapeutic memory is crucial for maintaining the therapeutic effects by applying the knowledge and skills gained from psychotherapy (Dong, Lee, and Harvey 2017). Previous studies have investigated the advantages of therapeutic metaphors in promoting insight and their neural correlates within the field of psychotherapy (Yu et al. 2019; Yu et al. 2021), as well as the advantages of insight in promoting memory and their neural correlates within the field of basic cognitive psychology (Chen et al. 2021; Kizilirmak et al. 2019; Kizilirmak et al. 2016; Ludmer, Dudai, and Rubin 2011). Metaphors have dual encoding advantages in long‐term memory (Paivio 1986). The present study will focus on the memory‐enhancing effects of therapeutic metaphors and their neural correlates, which have rarely been addressed in previous studies.

A metaphor can be described as a comparison that shows how two things that are not alike in most explicit ways are similar in another important implicit way. Therapeutic metaphors play an important role in delivering new (healthy) thought schemas in cognitive behavioral therapy (CBT) (Muran and Digiuseppe 1990). Metaphors have been used to observe the advantages of therapeutic insight and long‐term memory in past studies. First, insight is an important event in psychotherapy (Castonguay and Hill 2007). Metaphors help clients gain insights and generate therapeutic changes (Hu et al. 2018; Hu et al. 2023). Second, Martin and colleagues examined four dyads of experiential psychotherapy conducted in a naturally psychotherapeutic context and found that clients tended to recall therapists’ intentional metaphors, and it was more helpful than recalling other therapeutic events (Martin, Cummings, and Hallberg 1992). Hu et al. (2023) used a rigorous randomized controlled trial experimental design, in which 94 participants at risk of generalized anxiety disorder were randomly assigned into either a metaphorical CBT invention, a literal CBT invention, or a no‐intervention control. They found that participants in the metaphorical CBT group have larger improvements in anxiety symptoms and better memory about the intervention than those in the literal CBT group. These studies suggested that the use of metaphors during psychotherapy could enhance clients’ encoding and recall of important therapeutic events. However, their measurements of memory were subjective and rough, and their paradigms of clinical intervention were not suitable for investigating the brain activity in metaphor processing. Third, metaphorical micro‐counseling dialogs (MCDs) have been employed as experimental materials to observe the neural mechanisms of therapeutic metaphor processing and its correlation with insight experiences due to its advantages in presenting multiple therapeutic events in a limited time. In this paradigm, participants first read a description of a mental distress problem, then followed by a matched metaphorical, literal, or problem‐restatement solution. Previous studies have found that metaphor solutions provided to mental distress problems reliably induce stronger insight experiences (Yu et al. 2016), larger negative emotion improvement (Hu et al. 2018; Zhang et al. 2023), and are accompanied by stronger activations in the hippocampus, amygdala, thalamus, and dorsal striatum than literal solutions (Yu et al. 2019; Yu et al. 2021; Zhang et al. 2023). The hippocampus was proposed to break thinking fixations and form novel associations in insight processing and serve as a predictor of negative emotion improvement, and the amygdala was responsible for emotional arousal and strengthening memory consolidation with the hippocampus (Yu et al. 2019; Yu et al. 2021; Zhang et al. 2023). The above evidence showed that therapeutic metaphors containing insightful and emotional information would promote long‐term memory. However, the memory effects of therapeutic metaphors and their neural correlates were hardly studied in previous studies. Therefore, the present study aimed to investigate the effects of therapeutic metaphors on memory encoding and their neural correlates using metaphorical MCDs.

Therapeutic metaphors could mobilize multiple memory systems in long‐term memory encoding, each with distinct properties and neural bases (Ferbinteanu 2019). First, therapeutic metaphors promote episodic memory encoding. Dural‐coding theory of metaphor (Paivio 1986) proposed that the mental imagery system constructs a synchronously organized and comprehensive information structure that can store information in chunks, and the verbal system deeply integrates information through logical processing; they together improve long‐term memory. Providing metaphorical solutions to mental distress problems would create mental imagery with psychotherapeutic significance, therefore recruiting the hippocampus and parahippocampal gyrus, which were responsible for visuospatial processing and episodic memory encoding (Liang and Preston 2015). Second, positive experiences of insight produced by therapeutic metaphors would boost emotional memory encoding. Whether a representational change results in a goal state of insight mainly depends on the distance between the current and goal state in the new representation (Ohlsson 1984). Therapeutic metaphors provided more distant source concept representations from mental distress problems than literal solutions, therefore producing stronger insight experiences. The amygdala is responsible for emotional experiences of insight induced by therapeutic metaphors (Yu et al. 2019; Yu et al. 2021). Third, metaphor comprehension may be closely related to implicit/procedural memory encoding. The declarative‐procedural (DP) model (Ullman 2001, 2004) claims that the mental grammar that subserves the rule‐governed combination of lexical items into complex representations depends on a neural system of procedural memory that includes a network of basal ganglia and cerebellar structures, which supports the learning of motor and cognitive skills. Metaphor comprehension may partly depend on understanding implicit relational processing (Holyoak and Stamenković 2018), is supported by implicit learning (Drouillet et al. 2018), and could also recruit activities of the dorsal striatum (Desai 2022). Thus, metaphor representation uses the implicit rules of analogy by comparing the source and target concepts and then recruiting the neural network of implicit/procedural memory. Fourth, previous studies have found that the reactivity of multiple memory systems, such as the hippocampus, amygdala (Malejko et al. 2017), and basal ganglia (Barsaglini et al. 2014), could predict therapeutic changes of CBT. Furthermore, impaired functions within these systems can be improved following psychotherapy, drug therapy, or neurostimulation therapy (Battaglia, Di Fazio, and Battaglia 2023; Battaglia et al. 2024; Battaglia, Nazzi, and Thayer 2023; Hanuka et al. 2022; Moody et al. 2017). Especially for metaphor, our previous study has found that hippocampal activity associated with the processing of metaphorical solutions to self‐selected problems can predict increases in self‐efficacy and decreases in psychological distress (Zhang et al. 2023).

The subsequent memory paradigm was used to observe successful memory encoding of therapeutic metaphors and their neural activities. Several functional magnetic resonance imaging (fMRI) studies investigated neural correlates of successfully encoding insight events with this paradigm (Chen et al. 2021; Kizilirmak et al. 2019; Kizilirmak et al. 2016; Ludmer, Dudai, and Rubin 2011). In this paradigm, insight tasks were usually encoded during fMRI scanning, and then a memory retrieval test was performed 24 h (Kizilirmak et al. 2019; Kizilirmak et al. 2016) or 7 days later (Chen et al. 2021; Ludmer, Dudai, and Rubin 2011) outside the scanner. Learning trials were typically classified and compared based on whether the encoded items were subsequently remembered or forgotten. Most studies observed stronger amygdala activation in the contrast of remembered insight versus forgotten insight events by using the perceptual insight task (Ludmer, Dudai, and Rubin 2011) or the compound remote associates task (Kizilirmak et al. 2019; Kizilirmak et al. 2016). However, the hippocampus, a brain region typically involved in episodic memory encoding, was not found. One reason may be that these studies did not provide evidence of interaction analyses between remembered versus forgotten and insight versus non‐insight events. Recently, Chen et al. (2021) applied the task of Chinese *chengyu* riddles and found stronger hippocampus activity in the interaction analyses. This study aimed to investigate the advantage of therapeutic metaphors in memory encoding and their neural correlates by using metaphorical MCDs as materials and including experimental conditions of remembered and forgotten metaphors and remembered and forgotten literals, which allowed us to analyze the interaction effect. These efforts would contribute to obtaining reliable evidence of the strong memory‐encoding effect of therapeutic metaphors.

This study focused on neural mechanisms of successfully encoding therapeutic metaphors in mental health contexts. The encoding phase was performed during scanning. Subjects were first presented with a mental distress problem and then presented with the relevant metaphor or literal solution. They were asked to imagine themselves as clients in an interactive counseling scenario and to rate the insightfulness of solutions. The memory testing phase was conducted 1 week later outside the scanner. We hypothesized that (1) metaphor solutions would be remembered better than literal solutions, (2) metaphor solutions could mobilize multiple memory encoding systems such as episodic memory supported by the hippocampus and parahippocampal gyrus, emotional memory system supported by the amygdala, and implicit learning system supported by the dorsal striatum, and (3) insight ratings and activations of the above brain areas in encoding could significantly predict the memory performances in testing.

## 2 Materials and Methods

### 2.1 Transparency and Openness

We reported how we determined our sample size, all data exclusions, manipulations, and measures in the study. All data were available at the Science Data Bank and can be accessed at https://doi.org/10.57760/sciencedb.07563. Behavioral data were analyzed with the software of SPSS18, image data were analyzed with the software of statistical parametric mapping (SPM8, http://www.fil.ion.ucl.ac.uk/spm/), and regression analyses were performed with hierarchical linear modeling (HLM7). This study was not preregistered.

### 2.2 Participants

Twenty‐two undergraduate and fifteen graduate students from 26 majors of 19 universities in Beijing volunteered to participate in this study. All participants were right‐handed and native Chinese speakers from 25 cities in 12 provinces and were excluded if they reported having any medical, neurological, or psychiatric illnesses. Psychiatric illnesses were excluded based on participants’ self‐reports of a history of mental disorders and a preliminary assessment using the Patient Health Questionnaire‐9 (PHQ‐9). One participant was excluded due to psychiatric illness (reported a history of mental disorders and intense thoughts of suicidal activities or self‐harm in the PHQ‐9). Two participants were excluded because of identical hit rates and false alarm rates for solutions in the memory testing phase. Three participants were eliminated due to low numbers of remembered metaphor solutions (or trials; < 20%), which did not allow for meaningful fMRI data analysis. The remaining 31 participants (16 males and 15 females, 29 Han Chinese and 2 Manchu) had an average age of 21.42 years (standard deviation = 1.93), ranging from 18 to 24 years. All participants provided written informed consent and received financial compensation for their participation. This study was approved by the ethical guidelines of Hebei Normal University.

The sample size was determined based on previous fMRI studies on insight memory effects and calculated using G*Power 3.1. Previous studies obtained significant results with a sample size of 16–26 (Chen et al. 2021; Kizilirmak et al. 2019; Kizilirmak et al. 2016; Ludmer, Dudai, and Rubin 2011). A total of 27 subjects were required to detect an effect size as medium as *f* = 0.3 with a type I error of 5% and 85% power in the within‐subjects analysis of variance (ANOVA). This suggested that the sample size in the present study was appropriate.

### 2.3 Materials

This study included 72 items for the formal experiment. To familiarize participants with the task and procedure, four items were used for practice. Each item included a mental distress problem, one metaphorical or literal solution to the problem, one target, and four alternative theme words for the problem (see examples in Table 1). Mental distress problems and solutions were all chosen from the material database of metaphorical MCDs (Yu et al. 2016). A word that reflected the typical event was extracted from each text description of mental distress problems and called the “target theme word” (further details were provided in the Supporting Information). Thus, a list of “target theme words” for 72 items was built. For each item, there was only one correct target theme word out of four options; the other three alternatives were unrelated to the mental distress problem and randomly selected from the theme word list to ensure that the chance of selection was equal for each theme word. Finally, 36 metaphorical and 36 literal solutions were randomly allocated to 72 problems for each participant.

**TABLE 1:** Examples of experimental materials.

Mental distress problems	Metaphor solutions	Literal solutions	Target theme words	Alternative theme words
I feel extremely frustrated because of beginning a major I dislike.	Success in life is not holding good cards but playing bad cards well.	Success mainly depends on effort, and it is important to do a good job now.	Major	1. Trick 2. Love 3. Major 4. Try
I feel angry when I persuade those who dress vulgarly, because they do not accept my advice.	The shoe that fits one pinches another, there is no recipe for living that suits all cases.	Everyone has his own aesthetic view. You need not ask others to agree with you.	Advice	1. Shame 2. Blow 3. Participation 4. Advice
I have no motivation to do anything before I get the result of an important exam.	Life is like riding a bike, you must keep moving to keep your balance.	Live your life with your normal rhythm, thus you will not feel confused.	Exam	1. Appreciation 2. Examination 3. Desire 4. Separation
In my life, I am often hesitating and afraid of making a wrong decision and regretting it.	When the ship goes out of the sea it knows how to adjust its direction, or else stay in the harbor forever.	Instead of hesitating and missing the chance, it is better to make a serious choice and devoted to it.	Hesitation	1. Hesitation 2. Try 3. Goal 4. Opportunity

### 2.4 Design and Procedure

The experiment included two phases. Similar to the previous fMRI studies conducted by Ludmer, Dudai, and Rubin (2011) and Chen et al. (2021), the encoding phase was conducted in the fMRI scanner, and the memory testing phase was performed in another quiet laboratory 1 week later outside the scanner. During encoding, participants were not informed that their memory would be tested later. Mental distress problems and solutions were presented randomly in an event‐related manner using the software E‐prime 2.0.

#### 2.4.1 Encoding Phase

**FIGURE 1:**
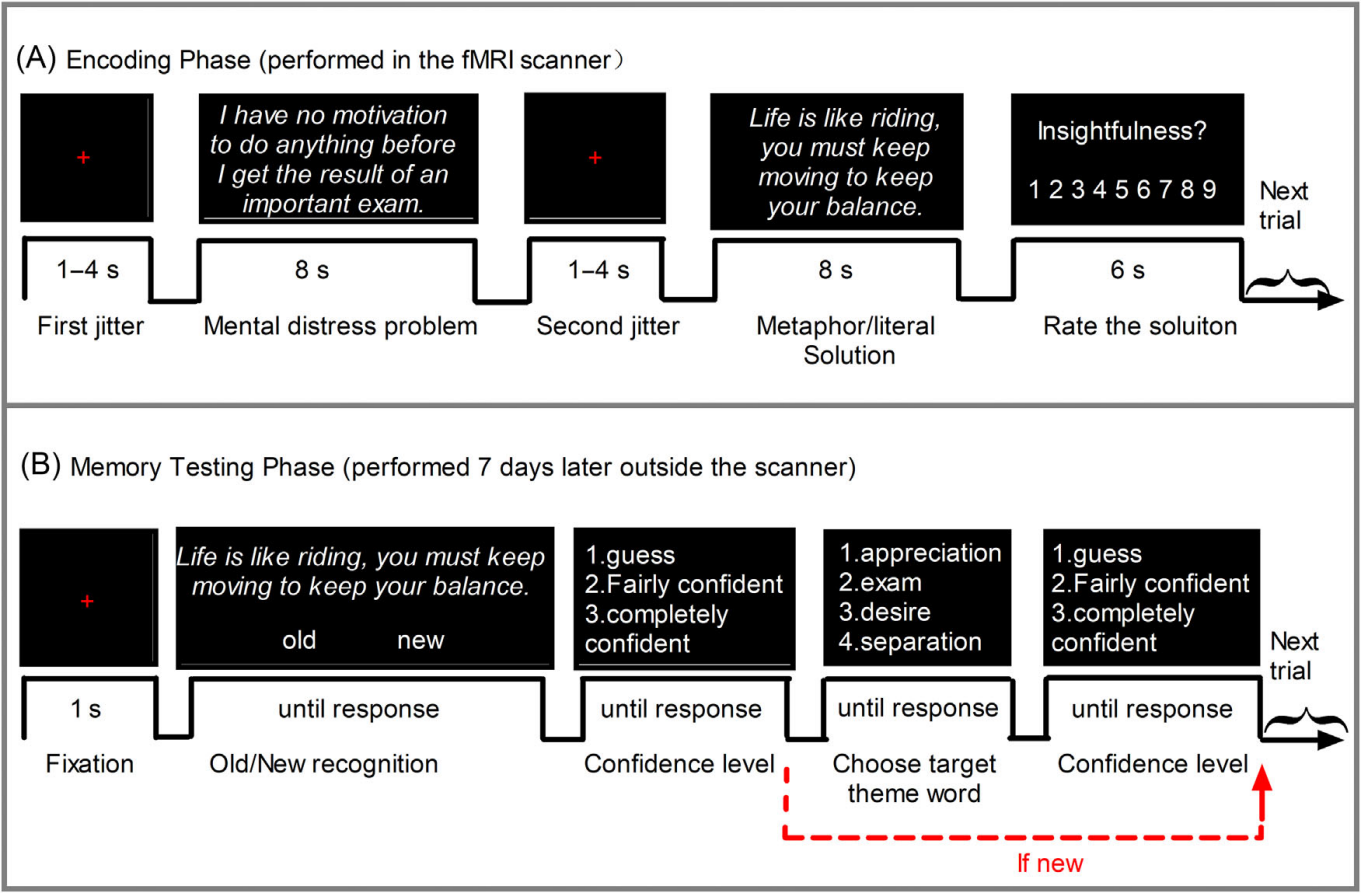
One exemplary trial used in the encoding and memory testing phase. (A) Encoding phase, performed in the scanner. A fixation cross presented for 1–4 s, followed by a mental distress problem with a fixed duration for 8 s. Then, another jitter was presented 1–4 s to allow for a separation of problem‐ and solution‐related brain responses, followed by a preallocated solution with a fixed duration of 8 s. Once solutions disappeared, participants were allowed 6 s to rate the insightfulness of solutions (i.e., to what extent there was a cognitive “click” or new enlightenment that improved understanding of a problem) on a nine‐point Likert scale (“1” meant not at all and “9” meant very much). (B) Memory testing phase, performed 7 days later outside the scanner. A fixation cross displayed for 1 s, then a solution was presented until participants judged it as old (presented during encoding) or new (not presented previously) by pressing the number “1” or “3” buttons (counterbalanced across participants). If the solution was recognized as old, then four theme words were presented until subjects chose the target theme word included in the mental distress problem by pressing the indicial number. Each decision was followed by a confidence rating on three levels (“1” meant guess, “2” meant fairly confident, and “3” meant completely confident).

The formal encoding phase was conducted in the scanner; 72 items were presented randomly in three blocks (12 metaphorical and 12 literal solutions randomly allocated per run). Within each block, different types of solutions were organized pseudo‐randomly and presented no more than three times in a row to avoid habituation and order effects. As shown in Figure 1A, in each trial, participants were instructed to first read a mental distress problem, then read a solution carefully with no other body action, and rate the insightfulness of the solution by pressing the response box. They were asked to imagine themselves as clients in an interactive counseling scenario that reflected what the sentences described vividly. The mental distress problem or solution was presented as a sentence in a line of white Chinese characters against a black background and projected onto the center of a screen through a mirror.

#### 2.4.2 Memory Testing Phase

The memory test included 72 old (solutions learned in the encoding phase) and 72 novel solutions (the remaining 36 metaphorical and 36 literal solutions not presented previously) and took place outside the scanner 7 days after the encoding phase was completed. These trials were randomly divided into three blocks, and 48 trials (12 old metaphorical, old literal, new metaphorical, and new literal solutions, respectively) were presented each block. As the example trial shown in Figure 1B, a solution was first presented until participants judged it as old or new, and if old, then four alternative theme words were presented until they chose the target theme word. Sequences of the four words were randomly presented to avoid order effects. Each decision was followed by a confidence rating as described by Ludmer, Dudai, and Rubin (2011).

### 2.5 Image Acquisition

Scanning sessions were conducted on a Siemens Trio 3.0 Tesla MR scanner at the Beijing MRI Centre for Brain Research with a 20‐channel head coil. The MRI session included three functional and one anatomical run for each participant. During the functional sessions, a blood oxygenation level‐dependent (BOLD) signal‐sensitive T2‐weighted echo‐planar imaging (EPI) sequence was acquired using a set of 32 interleaved axial slices to cover the entire brain (voxel size = 3 × 3 × 4 mm^3^, slice thickness = 4 mm, repetition time [TR] = 2000 ms, echo time [TE] = 30 ms, flip angle [FA] = 90°, field of view [FOV] = 192 × 192 mm^2^, and matrix size = 64 × 64 mesh). A high‐resolution T1‐weighted 3D magnetization‐prepared rapid gradient‐echo pulse sequence was acquired following the fMRI scan for the co‐registration and standardization to a template brain (voxel size = 1 × 1 × 1 mm^3^, slice thickness = 1 mm, TR = 2600 ms, TE = 3.02 ms, and FOV = 256 × 256 mm^2^).

### 2.6 Image Analyses

Image data were preprocessed and analyzed using SPM8. Functional images were first corrected for slice timing and head motion (scans with head movement larger than 2.5 mm or 2.5° in any direction were eliminated), followed by coregistration with their anatomical scans and spatial normalization using DARTEL. EPIs were then smoothed with a full‐width half‐maximum (FWHM) Gaussian kernel of 8 mm. A high‐pass filter with a cut‐off frequency of 1/128 Hz was applied to remove low‐frequency signal drifts.

For the first‐level analyses, two general linear models (GLMs) were estimated. The first GLM was estimated to observe which brain activation was involved in successfully encoding metaphor solutions. Based on memory performances in the testing phase, each solution learned in the scanner was divided into either later‐remembered (REM, solutions answered correctly both in the old/new recognition test for solutions and the target theme word recognition test for problems) or later‐forgotten (FOR) items, respectively. Therefore, four separate regressors were created in the first GLM: metaphor solutions later remembered (MET‐REM), metaphor solutions later forgotten (MET‐FOR), literal solutions later remembered (LIT‐REM), and literal solutions later forgotten (LIT‐FOR). The second GLM was estimated regardless of memory performance and included two separate regressors: metaphorical and literal solutions. This GLM was used to conduct regression analyses between brain activations of regions of interest (ROIs) in encoding and memory performances in testing. Events in these two GLMs were all time‐locked to the onset of solution presentation for 8 s and then convoluted using the canonical hemodynamic response function (HRF). Furthermore, motion realignment parameters were included to account for variance related to head movements, and serial correlations were corrected using an autoregressive (1) mode.

For the second‐level analyses, resulting contrast images from the first‐level analyses were subjected to two random‐effects analyses. Contrast images extracted from the first GLM were assessed using a 2 × 2 full factorial group ANOVA, in which solution types (metaphorical or literal) and memory (remembered or forgotten) were both set as within‐subject variables. Contrast images extracted from the second GLM were assessed using a paired‐sample *t*‐test. Bilateral parahippocampal gyrus, hippocampus, thalamus, amygdala, caudate, putamen, and cerebellum were used as ROIs because they were typical regions of episodic, emotional, and procedural/implicit memory encoding. A small volume correction (SVC) was applied to these ROIs with an anatomical mask according to the automated anatomical labeling template provided by the software of WFU PickAtlas 3.0 (http://fmri.wfubmc.edu/software/PickAtlas). For whole‐brain analyses, the threshold was set at *p *< 0.001 for voxel level (uncorrected), *p *< 0.05 for cluster level (uncorrected), and 50 or more contiguous voxels. For SVC analyses, the threshold was set at *p *< 0.001 for voxel level (uncorrected), *p *< 0.05 for cluster level (FWE corrected), and 10 or more contiguous voxels.

These ROIs were defined as spheres with a radius of 0.5 mm centered on the peak coordinates defined by the contrast of metaphor > literal, respectively. Regressions between activities of ROIs extracted from this contrast during encoding and memory performances during testing were performed with HLM. Memory performances (hit rate, false alarm rate, *Pr*, and accuracy of target theme words) were entered as the outcome variables, and activities of these ROIs were entered as predictor variables in the two‐level HLMs, respectively, with solution types (Level 1: metaphorical, literal) nested within participants (Level 2).

### 2.7 Behavioral Data Analyses

Signal detection theory was used to scale recognition memory performance. *Pr* was calculated by subtracting the false alarm rate from the hit rate. Two analyses were conducted to examine relationships between insight scores rated in the encoding phase and recognition memory performances in the testing phase. First, paired‐sample *t*‐tests were performed between the remembered and forgotten separately for metaphor and literal solutions. Second, memory performances were entered as outcome variables, and the insight score was entered as the predictor variable in the two‐level HLMs, respectively.

## 3 Results

### 3.1 Behavioral Results

Descriptive statistics of insight scores in the encoding phase and memory performances in the testing phase are presented in Table S1.

#### 3.1.1 Insight Scores in the Encoding Phase

**FIGURE 2:**
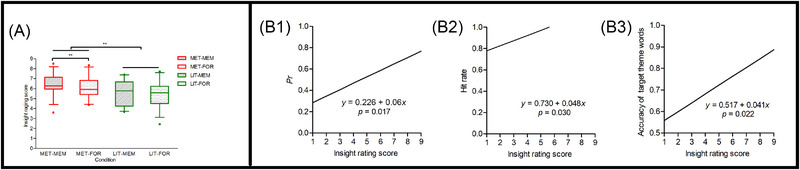
Relationships between the insight scores in encoding and memory performances in testing. (A) Box and whisker plots show the 5th, 25th, 50th (median), 75th, and 95th percentiles of insight rating scores of solutions in the encoding phase. Insight scores of metaphor solutions were significantly higher than those of literal solutions regardless of memory performances, and insight scores of the later remembered were higher than those of the later forgotten for metaphor solutions. **p < 0.01. The lines displayed in the B1 (*Pr*), B2 (hit rate), and B3 (accuracy of target theme words) were schematic diagrams to show regressions between insight scores in encoding and memory performances in testing. MET‐FOR = metaphor solutions later forgotten, MET‐MEM = metaphor solutions later remembered, LIT‐FOR = literal solutions later forgotten, and LIT‐REM = literal solutions later remembered.

Regardless of memory performances, insight scores of metaphor solutions were significantly higher than those of literal solutions, *t*(30) = 3.317, *p *= 0.002, *d* = 0.596, 95% CI = [0.209, 0.974] (Table S1 and Figure 2A), indicating that metaphor solutions were reliable in inducing strong insight experiences in mental health contexts.

#### 3.1.2 Memory Performances in the Testing Phase

The accuracy of target theme words for metaphor and literal solutions was 0.438 and 0.502 (Table S1), respectively, indicating that the numbers of remembered and forgotten trials were sufficient to allow for meaningful fMRI contrasts for metaphor and literal items.

*Pr* and the recognition confidence of metaphor solutions were both significantly higher than those of literal solutions, *t*(30) = 8.908, *p* < 0.001, *d* = 1.600, 95% CI = [1.059, 2.129], and *t*(30) = 5.817, *p* < 0.001, *d* = 1.045, 95% CI = [0.599, 1.479], respectively (Table S1).

#### 3.1.3 Relationships Between Insight Scores in the Encoding Phase and Memory Performances in the Testing Phase

First, insight scores of the remembered were higher than those of the forgotten metaphor solutions, *t*(30) = 2.963, *p* = 0.006, *d* = 0.532, 95% CI = [0.152, 0.905], whereas there was no difference between the remembered and forgotten literal solutions, *t*(30) = 1.082, *p = *0.288, *d* = 0.194, 95% CI = [−0.163, 0.548] (Table S1 and Figure 2A). Second, insight scores of solutions rated in the encoding phase positively predict memory performances in the testing phase: *Pr*, *β = *0.06, *p* = 0.017 (Figure 2B1); hit rate, *β = *0.048, *p* = 0.030 (Figure 2B2); accuracy of target theme words, *β = *0.041, *p* = 0.022 (Figure 2B3), respectively.

### 3.2 Imaging Results

#### 3.2.1 Neural Correlates of Processing Metaphor Solutions in the Encoding Phase

The contrast of metaphor > literal indicated that widespread brain areas showed greater activation in processing metaphor solutions relative to literal solutions, including the dorsal and lateral frontal gyrus, inferior frontal gyrus, inferior/middle/superior temporal gyrus, medial temporal gyrus, thalamus, amygdala, dorsal striatum, and cerebellum (see details in Table S2 and Figure S1).

#### 3.2.2 Neural Correlates of Remembered and Forgotten Metaphor Solutions

**FIGURE 3:**
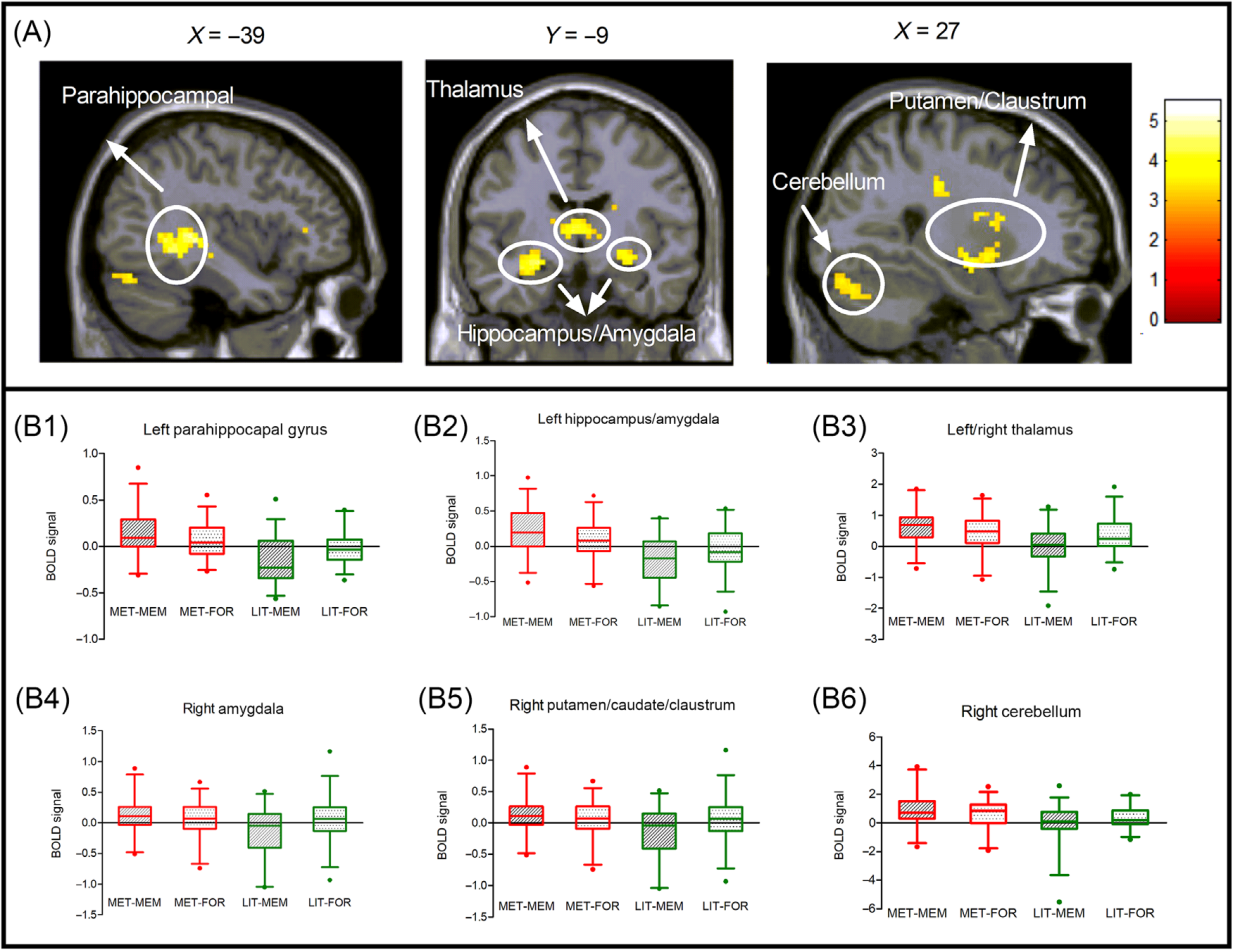
Imaging results for remembered and forgotten metaphor solutions identified in the interaction analysis. (A) Brain regions activated in the interaction analysis of ([MET‐REM]&amp;#x000A0;−&amp;#x000A0;[LIT‐REM])&amp;#x000A0;>&amp;#x000A0;([MET‐FOR]&amp;#x000A0;−&amp;#x000A0;[LIT‐FOR]). BOLD signals activated in the (B1) left parahippocampal gyrus, (B2) left hippocampus/amygdala, (B3) bilateral thalamus, (B4) right amygdala, (B5) right putamen/caudate/claustrum, and (B6) right cerebellum. Box and whisker plots show the 5th, 25th, 50th (median), 75th, and 95th percentiles of BOLD signals activated in the regions found in the interaction analysis with a threshold set at *p *< 0.001 for voxel level (uncorrected), *p* < 0.05 for cluster level (uncorrected), and 50 or more contiguous voxels. MET‐FOR = metaphor solutions later forgotten, MET‐MEM = metaphor solutions later remembered, LIT‐FOR = literal solutions later forgotten, and LIT‐REM = literal solutions later remembered.

The interaction analysis of ([MET‐REM] − [LIT‐REM]) > ([MET‐FOR] − [LIT‐FOR]) identified regions of the parahippocampal gyrus, hippocampus, thalamus, amygdala, putamen, caudate, claustrum, and cerebellum (Table 2 and Figure 3). The contrast of [MET‐REM] > [LIT‐REM] identified the dorsal and lateral frontal gyrus, inferior frontal gyrus, inferior/middle/superior temporal gyrus, hippocampus, parahippocampal gyrus, thalamus, amygdala, putamen, caudate, and cerebellum; the contrast of [MET‐FOR] > [LIT‐FOR] identified the dorsal and lateral frontal gyrus, inferior frontal gyrus, inferior/middle/superior temporal gyrus, hippocampus, parahippocampal gyrus, thalamus, and cerebellum (Table 2). No suprathreshold clusters were identified in the contrasts of [MET‐REM] > [MET‐FOR] and [LIT‐REM] > [LIT‐FOR] (Table 2).

**TABLE 2:** Neural correlation of remembered and forgotten metaphor solutions.

			MNI coordinates			Talairach coordinates				
Brain areas	BA	Cluster size	*x*	*y*	*z*	*x*	*y*	*z*	*T*	*Z*
**([MET‐REM] − [LIT‐REM]) > ([MET‐FOR] − [LIT‐FOR])**										
Left parahippocampal gyrus	19	121	−39	−48	−6	−39	−47	−3	4.46	4.28
Left amygdala		91	−24	−9	−12	−24	−9	−10	4.51	4.33
Left hippocampus			−33	−9	−15	−33	−9	−12	4.09	3.95
Left parahippocampal gyrus	28		−21	−27	−12	−21	−27	−9	3.56	3.47
Left thalamus		69	0	−12	9	0	−11	9	4.49	4.31
Right thalamus			12	−3	6	12	−3	6	3.49	3.40
Right putamen/caudate		79	27	−6	−9	27	−6	−7	4.04	3.90
Right claustrum			30	3	−9	30	3	−8	4.02	3.89
Right amygdala			30	0	−15	30	−1	−13	4.00	3.87
Right declive		78	30	−84	−24	30	−82	−16	3.60	3.50
Right uvula			33	−69	−33	33	−68	−24	3.38	3.30
Left putamen^a^		12	−27	−9	−9	−27	−9	−7	3.95	3.83
**[MET‐REM] > [LIT‐REM]**										
Left fusiform gyrus	37	10518	−45	−57	−12	−45	−56	−7	9.29	Inf
Left inferior/lateral frontal gyrus	46/9		−45	30	15	−45	30	12	8.31	7.37
Left hippocampus			−36	−12	−15	−36	−12	−12	7.77	6.98
Left superior temporal gyrus	41		−42	−36	0	−42	−35	2	7.55	6.82
Left inferior temporal gyrus	20		−42	−15	−21	−42	−15	−17	7.22	6.57
Left amygdala			−24	−9	−12	−24	−9	−10	6.96	6.36
Right inferior/lateral frontal gyrus	46/9		54	33	24	53	33	20	7.23	6.57
Right fusiform gyrus	3		45	−51	−12	45	−50	−8	6.79	6.23
Left superior frontal gyrus	9/8/6	461	−6	57	39	−6	57	33	5.35	5.05
Right superior frontal gyrus	6		3	6	66	3	9	60	4.38	4.21
Right amygdala^a^		29	30	−6	−15	30	−6	−12	5.09	4.83
Right hippocampus^a^		47	39	−15	−21	39	−15	−17	4.58	4.39
Left parahippocampal gyrus^a^		24	−33	−27	−18	−33	−27	−14	5.43	5.12
Left thalamus^a^		286	−9	−9	12	−9	−8	11	5.74	5.38
Right thalamus^a^		158	3	−15	6	3	−14	6	4.94	4.70
Left putamen^a^		175	−27	−9	−9	−27	−9	−7	6.62	6.10
Right putamen^a^		14	30	−9	−9	30	−9	−7	4.49	4.31
Left caudate^a^		123	−12	−6	15	−12	−5	14	5.43	5.12
Right caudate^a^		80	12	0	12	12	1	11	4.53	4.35
Left cerebellum^a^		107	−6	−78	−21	−6	−76	−14	4.79	4.57
Right cerebellum^a^		151	15	−78	−27	15	−77	−19	6.68	6.15
**[MET‐FOR] > [LIT‐FOR]**										
Left inferior/lateral frontal gyrus	46/9/6	2208	−42	27	18	−42	27	15	8.77	7.69
Left fusiform gyrus	37		−45	−57	−12	−45	−56	−7	7.91	7.08
Left middle/inferior temporal gyrus	21		−57	−48	−3	−56	−47	0	6.13	5.71
Left hippocampus			−36	−21	−18	−36	−21	−14	5.13	4.86
Left middle occipital gyrus	18		−30	−90	−9	−30	−88	−3	5.06	4.81
Left superior temporal gyurs	22		−60	−27	3	−59	−26	4	4.78	4.56
Left parahippocampal gyrus	36		−36	−36	−15	−36	−36	−11	4.16	4.01
Right middle frontal gyrus	46/9	493	51	36	18	50	36	15	5.61	5.27
Right middle/inferior occipital gyrus	18	581	33	−87	0	33	−84	4	5.36	5.06
Right inferior temporal gyrus	37		42	−54	−12	42	−53	−7	5.07	4.82
Right middle temporal gyrus	22		66	−42	0	65	−41	2	4.96	4.72
Left thalamus		114	−6	−27	−3	−6	−26	−1	4.89	4.66
Right cerebellum^a^		13	12	−75	−27	12	−74	−19	3.87	3.75

*Note*: Only clusters (with local maxima coordinates) up to the thresholds of *p* < 0.001 for voxel level (uncorrected), *p* < 0.05 for cluster level (uncorrected), and 50 or more contiguous voxels were reported. Clusters up to the thresholds of *p* < 0.001 for voxel level (uncorrected), *p* < 0.05 for cluster level (FWE corrected), and 10 or more contiguous voxels were reported.

Abbreviation: BA, Brodmann area.

^a^ Small volume correction.

#### 3.2.3 Relationship Between Brain Activation of Metaphor Encoding and Memory Performance

**FIGURE 4:**
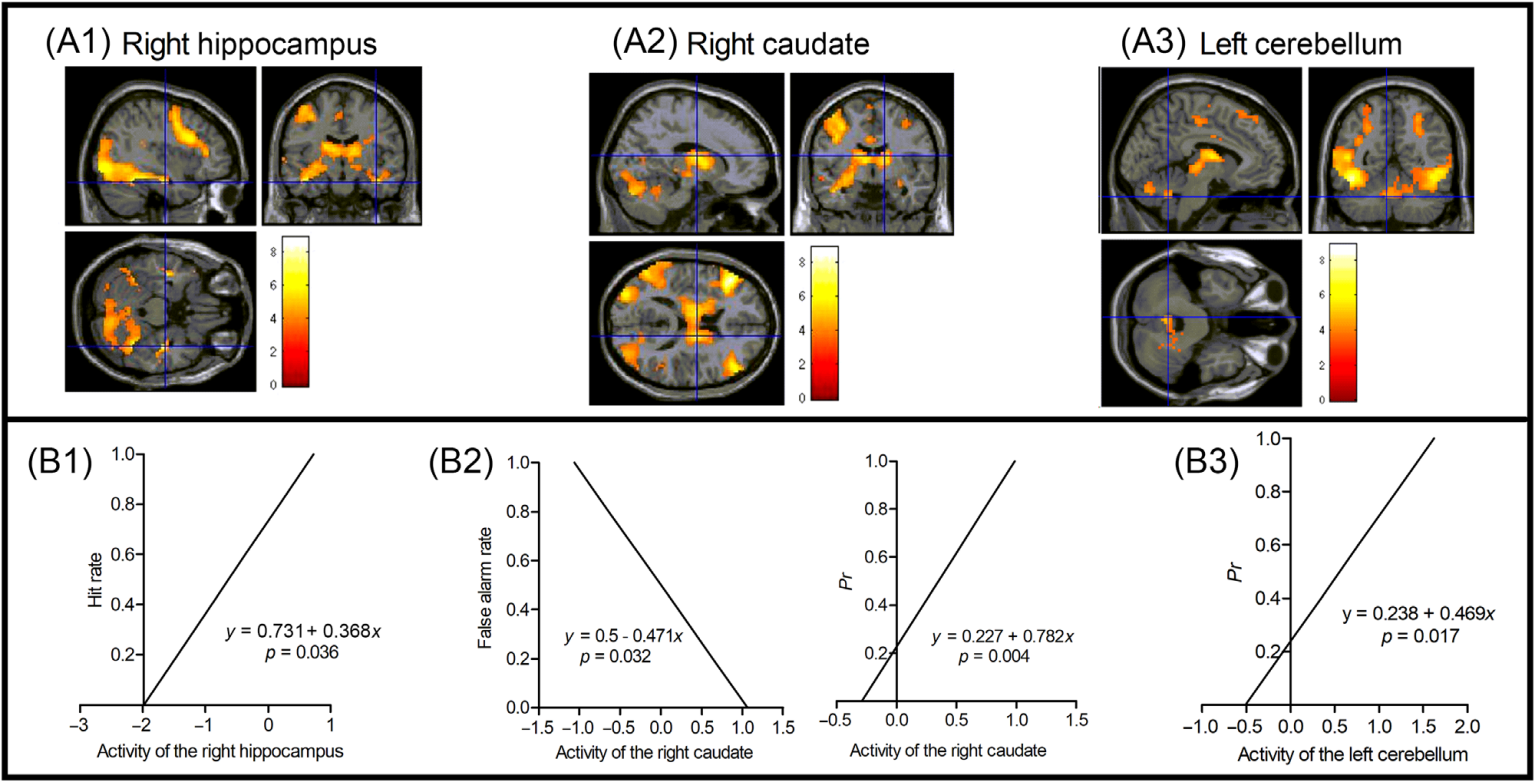
Regressions between neural activations of metaphor encoding and memory performances. The peak MNI coordinates of the (A1) right hippocampus (39, −12, −24), (A2) right caudate (15, −6, 15), and (A3) left cerebellum (−6, −54, −33) activated in the contrast of Metaphor > Literal. Activities of the right hippocampus (B1), right caudate (B2), and left cerebellum (B3) significantly predicted memory performances. The lines were schematic diagrams to show regressions between brain activities in the encoding phase and memory performances in the testing phase.

Multilevel regressions between the activities of ROIs in encoding and memory performances in testing were analyzed with HLM. The results revealed that activities of the right hippocampus (MNI [39, −12, −24], hit rate, *β = *0.368, *p* = 0.036), right caudate (MNI [15, −6, 15], *Pr*, *β = *0.782, *p* = 0.004; false alarm rate, *β = *−0.471, *p* = 0.032, respectively), and left cerebellum (MNI [−6, −54, −33], *Pr*, *β = *0.469, *p* = 0.017) significantly predict memory performances (Table S3 and Figure 4). Regression coefficients between activities of the parahippocampal gyrus, thalamus, amygdala, putamen, and memory performances were not significant (all *p*s > 0.05; see details in Table S3).

## 4 Discussion

This study used metaphorical MCDs to investigate the promoting effects of therapeutic metaphors on memory and their neural mechanisms. Metaphor solutions had stronger insight experiences and better memory performances than literal solutions, and their memory encoding was supported by multiple memory systems, including the parahippocampal gyrus, hippocampus, thalamus, amygdala, dorsal striatum, and cerebellum.

### 4.1 Metaphor Solutions Were Associated With Stronger Insight Experiences and Better Memory Performances

In line with our first hypothesis, recognition performances of metaphor solutions were significantly higher than those of literal solutions. These results were consistent with previous studies on the insight memory effect (Danek and Wiley 2020; Du et al. 2017), and this study was the first to extend this effect into a psychotherapeutic scenario. Furthermore, the strength of insightfulness produced by metaphor solutions in encoding positively predicted the recognition performance of solutions in memory testing, which supported the third hypothesis and was consistent with the findings by Danek and Wiley (2020). They found that the amount of “aha” experiences assessed during insight predicts later recall of solutions in a magic trick task. This evidence suggests that metaphor‐based psychotherapeutic information can last longer.

### 4.2 Multiple Memory Systems of Successfully Encoding Metaphor Solutions

fMRI interaction analyses showed that successful encoding of metaphor solutions was associated with the episodic memory system, including the entorhinal cortex (BA28), posterior parahippocampal (BA19), hippocampus, and thalamus; the emotional memory system, including the bilateral amygdala; and the procedural/implicit memory system, including the caudate, putamen, claustrum, and cerebellum. These regions were also found in the contrast of remembered metaphor versus remembered literal. These findings supported the second hypothesis.

#### 4.2.1 Episodic Memory Encoding System

Activations of the anterior hippocampus, entorhinal cortex, posterior parahippocampal areas, and thalamus were found in the interaction analyses; these subregions may be involved in episodic memory encoding of metaphor solutions in two possible ways (Aggleton and O'Mara 2022; Baumann and Mattingley 2016; Liang and Preston 2015). First, the posterior parahippocampal gyrus could be associated with episodic memory by visuospatial processing (Baumann and Mattingley 2016) and imagery encoding proposed by the dural‐coding theory of metaphor (Paivio 1986). Metaphor solutions have inherent concrete and imaginative features; thus, imagery visuospatial encoding of metaphor leads it to be recalled more easily than literal solutions. Second, the hippocampus and entorhinal cortex were active in context processing (Baumann and Mattingley 2016; Smith and Bulkin 2014) and played complementary roles in supporting episodic memory. The thalamus has key cognitive functions in the contextual encoding of episodic memory (Aggleton and O'Mara 2022). Consistently, this study used problem solutions to arouse personal problematic contextual experiences, and this context processing was critical to enhance the insight impact of therapeutic metaphor (Yu et al. 2021). In conclusion, the hippocampus, entorhinal cortex, posterior parahippocampal, and thalamus were deeply involved in episodic memory encoding of therapeutic metaphor.

In this study, the results of the interaction and regression analyses both indicated that the hippocampus plays a critical role in successfully encoding metaphor/insight solutions. These results were consistent with the findings by Chen et al. (2021). However, other studies on insight memory advantage found the amygdala rather than the hippocampus responsible for the successful encoding of insight events (Kizilirmak et al. 2019; Kizilirmak et al. 2016; Ludmer, Dudai, and Rubin 2011). One reason could be that these studies couldn't analyze the interaction effect between remembered versus forgotten and insight versus non‐insight events. Specifically, the non‐insight items were not involved in the memory testing due to their unsolvability (Kizilirmak et al. 2019), or the number of remembered non‐insight items was insufficient for meaningful fMRI analysis, partly due to the design of the task and the interval between encoding and retrieval (Kizilirmak et al. 2016; Ludmer, Dudai, and Rubin 2011). Therefore, they could only capture differences in emotional memory encoding by making fMRI contrasts between remembered and forgotten insight items. In contrast, the interaction analysis completely included insight/non‐insight and remembered/forgotten conditions; therefore, it could capture differences both in episodic and emotional memory encoding. The present study and Chen et al. (2021) both realized the interaction analyses and found the hippocampus activity, indicating the co‐involvement of episodic and emotional memory encoding for remembered metaphor/insight items.

#### 4.2.2 Emotional Memory Encoding System

This study found increased amygdala activities and insight experiences in the interactive analyses, which were consistent with previous studies on insight‐promoting memory (Chen et al. 2021; Kizilirmak et al. 2019; Kizilirmak et al. 2016; Ludmer, Dudai, and Rubin 2011). Metaphor solutions to mental distress problems were associated with stronger insight experiences, more positive emotionality, and higher amygdala activities than literal solutions (Yu et al. 2019; Yu et al. 2021; Yu et al. 2016). This positive experience directly contributed to emotional memory encoding. A meta‐analysis study of successful emotional encoding revealed that the amygdala was reliably engaged in human emotional memory encoding, interacted with the hippocampus and prefrontal lobe to promote perceptual processing, semantic elaboration, and attention, and then contributed to subsequent memory of emotional material (Murty et al. 2010). Therefore, we suggested that the amygdala plays an important role in encoding positive emotions of insight experiences and modulating strengthened emotional memory consolidation along with the hippocampus (Yu et al. 2021).

#### 4.2.3 Procedural/Implicit Memory Encoding System

This study found activations of the caudate, putamen, claustrum, and cerebellum in the interaction analyses, and activations of ROIs in the caudate and cerebellum during encoding could predict long‐term memory performances in the multilevel regression analyses. These findings may reflect implicit/procedural learning features of therapeutic metaphors. First, the DP model (Ullman 2001, 2004) claims that the mental lexicon of memorized word‐specific knowledge depends on the largely temporal‐lobe substrates of declarative memory, which underlie the storage and use of knowledge of facts and events. The mental grammar, which subserves the rule‐governed combination of lexical items into complex representations, depends on a neural system of procedural memory. This system underlies the learning and execution of motor and cognitive skills and is composed of a network of specific frontal, basal ganglia, parietal, and cerebellar structures. Recent meta‐analyses show that the process of language generation and understanding recruits the neural system of procedural memory and supports the DP model (Bulut and Hagoort 2024; Laura et al. 2021). Metaphor is regarded as a factor determining syntactical structure (Kuzmina 2013). Its implicit meanings must be comprehended with the help of analogy and the mapping rules of mental grammar. Therefore, metaphorical language is more likely to activate the procedural memory system than literal language. Second, metaphors have a significant embodied basis, and when reading metaphorical language involving sensations and motors, brain regions associated with performing these sensorimotor memories are activated, such as the basal ganglia and cerebellum (Desai 2022). The engagement of procedural memory of sensorimotor systems suggests that metaphors not only evoke insight but also facilitate the internalization of therapeutic techniques in a manner similar to skill learning. Third, there is evidence indicating that metaphor comprehension is related to implicit processing. Metaphor comprehension grasps implicit abstract meanings by suppressing their explicit literal meanings, and the basal ganglia may be particularly active when a preferred interpretation is suppressed (Williams 2020). Metaphor comprehension may partly depend on understanding implicit relational processing (Holyoak and Stamenković 2018). Experimental evidence supports the relationship between implicit learning and metaphor (Drouillet et al. 2018) and the involvement of the basal ganglia in understanding metaphor (Desai 2022). Fourth, the encoding of therapeutic metaphors in the context of mental distress problem‐solving is a kind of complex learning. Improvements in the complex learning task reflected a combination of implicit and explicit learning and allowed their neurocognitive architecture to interact with each other (Reber 2013). For example, the hippocampus and dorsal striatum were found to be activated when the rats learned a complex task that included both spatial navigation and sequential training (Ferbinteanu 2016). The hippocampus and basal ganglia both support a multi‐step problem‐solving task with arbitrary and changing starting and desired ending states (Zarr and Brown 2023). The claustrum's extensive and reciprocal interconnectivity with most of the neocortex, as well as subcortical structures, may position it as the center of consciousness and unconsciousness processing (Liaw and Augustine 2023). This positioning facilitates the cooperation of hippocampal and striatal memory systems in complex learning conditions. Probably in a similar way, multiple memory systems, including the medial temporal gyrus, amygdala, dorsal striatum, and cerebellum, are cooperatively involved in the complex learning of therapeutic metaphors.

### 4.3 Neural Activities Related to Comprehending Rather Than Remembering Metaphors

Therapeutic metaphors also involve several mental processes other than memory encoding, such as semantic retrieval, comparison and similarity extraction, and assessment for insight value in mental distress problem‐solving. They could recruit brain networks related to the working memory system, such as the dorsolateral prefrontal cortex, and language processing system, such as the superior/middle temporal gyrus, including Wernicke's area, inferior frontal gyrus, and Broca's area (Ardila, Bernal, and Rosselli 2016). However, these areas were found both in the contrast of remembered metaphor versus remembered literal and that of forgotten metaphor versus forgotten literal, but not found in the interaction analysis. This meant that these areas contribute to metaphor comprehension but not to metaphor memory encoding.

### 4.4 Conclusions and Limitations

The present study suggested that therapeutic metaphors promote insight experiences and long‐term memory retention of solutions and engage multiple memory systems during encoding, including the episodic memory system based on the medial temporal gyrus, the emotional memory system based on the amygdala, and the procedural/implicit memory system based on the dorsal striatum and cerebellum. The findings had high ecological validity because the client‐therapist interactive micro‐talks closely resembled real‐world situations and had important clinical implications, as the memory of treatment can provide clients with the resources to face subsequent changes in daily life. On the one hand, it implied that the use of therapeutic metaphor should be emphasized in psychotherapy, as incorporating metaphors into therapy practices helps clients to think more deeply or elaborately about their distressed problems and to achieve more successful memory encoding of psychotherapy. On the other hand, it implied that non‐metaphorical therapeutic information might be better encoded if it could engage multiple memory neural systems, either by including strong episodic, emotional, or sensorimotor therapeutic content or by stimulating brain areas associated with encoding therapeutic metaphors using neuromodulation technology in clinical populations.

This study had several limitations. First, the amygdala was assumed to be responsible for the positive emotion of insight experiences, but the emotionality of solutions was not measured in this study. Second, the fMRI data were collected only for the memory encoding phase; neural activity during the memory retrieval phase remains unstudied. Third, the connection between the memory of metaphors and long‐term therapeutic change was not strongly argued. Future studies could adopt a randomized controlled trial to evaluate the connection between memory and therapeutic changes of metaphors in different populations, which may help us better understand how memory retention of metaphors affects therapeutic outcomes. Finally, participants in the present study were all highly educated university students from China, which may limit the generalizability of the findings. Although metaphors are often used to facilitate insight and memory retention, not all subjects may respond to or comprehend metaphors in the same way. Cultural differences or language‐specific effects in metaphor interpretation, or cognitive limitations in certain populations, might affect the efficacy of metaphor‐based intervention. As we have stated in the previous study (Yu et al. 2019), the same metaphor, such as using cards to interpret college majors, might have a different impact on individuals from different cultural backgrounds due to the limitation of cultural appropriateness. Our previous study indicated that the insight experience of therapeutic metaphor in major depressive disorder patients was different from those of healthy adults due to the deficits of cognitive control (Jiang et al. 2016). Although the current results were important for understanding the nature of metaphor‐promoting memory encoding in the field of psychotherapy, it may be cautious to infer this conclusion in clinical practice. Future studies could consider a broader range of individual variables that might influence findings to provide a more balanced view of the therapeutic use of metaphor.

## Author Contributions

**Fei Yu**: conceptualization, methodology, data curation, investigation, validation, formal analysis, project administration, visualization, funding acquisition, writing–original draft, writing–review and editing. **Zhijie Zhang**: conceptualization, funding acquisition, supervision, resources. **Wencai Zhang**: conceptualization, methodology, software, formal analysis, supervision, resources, writing–review and editing, validation.

## Conflicts of Interest

The authors declare no conflicts of interest.

## Ethics Statement

All of the participants provided written informed consent and received financial compensation for their participation. This study was approved by the ethical guidelines of Hebei Normal University.

### Peer Review

The peer review history for this article is available at https://publons.com/publon/10.1002/brb3.70270.

## Supporting information



Suppoting Information

## Data Availability

The data that support the findings of this study are openly available in Science Data Bank at https://doi.org/10.57760/sciencedb.07563.
