# Supplementary material for: Therapeutic Metaphors Enhance Memory Systems in Mental Health Contexts
Source: Brain Behav. 2025 Jan 19;15(1):e70270. doi: 10.1002/brb3.70270 (PMC11743976; doi:10.1002/brb3.70270)
Supplement: Supplementary file 1 — Suppoting Information [file BRB3-15-e70270-s001.docx]

***Supplementary information***

**Therapeutic Metaphors Enhance Memory Systems in Mental Health Contexts**

Fei Yu^a^, Zhijie Zhang^a^, and Wencai Zhang^b*^

^a^Department of Psychology, Hebei Normal University, No. 20 East Nanerhuan Road, Shijiazhuang 050024, P.R.China

^b^Key Laboratory of Mental Health, Institute of Psychology, Chinese Academy of Sciences (CAS), 16 LinCui Road, Chaoyang District, Beijing 100101, P.R.China

Correspondence concerning this article should be addressed to Wencai Zhang, Key Laboratory of Mental Health, Institute of Psychology, Chinese Academy of Sciences (CAS), 16 Lincui Road, Chaoyang District, Beijing 100101, China. Tel: +86 010 64855932. E-mail: [zhangwc@psych.ac.cn](mailto:zhangwc@psych.ac.cn)

**Supplemental Experimental Procedures**

**Pre-screening and Generation of the Target Theme Words**

Seventy-six different target theme words were first generated by the authors, and each of them was consisted of two Chinese characters that directly derived from the text descriptions of mental distress problems. For an example illustrated in Table 1, the target theme word ‘advice’ was extracted from the mental distress problem ‘I feel angry when I persuade those who dress vulgarly, because they do not accept my advice.’ Next, 76 descriptions of mental distress problems and related target theme words were evaluated by three graduate psychology students who would not participate in the formal fMRI experiment. They were asked to evaluate whether theses target theme words could reflect the main events involved in the corresponding descriptions of mental distress problems. If they thought these target theme words provided by us were inappropriate, then they were asked to extract another target theme words that were thought to be more appropriate for these mental distress problems. There were six target theme words evaluated as inappropriate by two or three rates, and then reformed overall consideration the theme words the raters provided.

**Supplemental Information for Results**

**Table S1** Insight Scores in the Encoding Phase and Memory Performances in the Testing Phase

| （*N* = 31） | Metaphor solutions | | Literal solutions | |
| --- | --- | --- | --- | --- |
|  | *M* | *SD* | *M* | *SD* |
| **Encoding phase** |  |  |  |  |
| Insight scores regardless of memory performances | 6.242 | 1.017 | 5.517 | 1.148 |
| Insight scores later-remembered | 6.439 | 1.085 | 5.578 | 1.187 |
| Insight scores later-forgotten | 6.080 | 1.069 | 5.448 | 1.256 |
| **Memory testing phase** |  |  |  |  |
| Hit rate | 0.727 | 0.160 | 0.712 | 0.178 |
| False alarm rate | 0.236 | 0.152 | 0.514 | 0.186 |
| Pr (hit rate r false alarm rate) | 0.491 | 0.170 | 0.204 | 0.098 |
| Reaction time for old/new recognition (ms) | 4713.208 | 1675.066 | 5071.182 | 2054.251 |
| Confidence of old/new recognition | 2.312 | 0.282 | 2.065 | 0.297 |
| Accuracy of target theme words | 0.438 | 0.147 | 0.502 | 0.151 |

*M*, mean; *SD*, standard deviation.

**Table S2** Activation Clusters for Processing Metaphor Solutions Identified in the Contrast of Metaphor > Literal

| Brain areas | BA | Cluster size | MNI Coordinates | | | Talairach Coordinates | | | t | Z |
| --- | --- | --- | --- | --- | --- | --- | --- | --- | --- | --- |
|  |  |  | x | y | z | X | y | z |  |  |
| Left Inferior Temporal Gyrus | 19 | 9698 | -45 | -63 | -9 | -45 | -61 | -4 | 8.90 | 6.18 |
| Left Inferior Frontal Gyrus | 46 |  | -45 | 30 | 12 | -45 | 30 | 10 | 8.31 | 5.94 |
| Left Fusiform Gyrus | 37/20 |  | -42 | -51 | -15 | -42 | -50 | -10 | 7.90 | 5.77 |
| Left Parahippocampal Gyrus | 36 |  | -39 | -27 | -15 | -39 | -27 | -11 | 7.07 | 5.38 |
| Left Sub-Gyral | 20/37 |  | -42 | -15 | -21 | -42 | -15 | -17 | 6.99 | 5.35 |
| Right Inferior Frontal Gyrus | 46 |  | 48 | 36 | 12 | 48 | 35 | 9 | 6.88 | 5.29 |
| Right Inferior Occipital Gyrus | 19 |  | 42 | -78 | -12 | 42 | -76 | -6 | 6.57 | 5.13 |
| Left Middle Occipital Gyrus | 19 |  | -30 | -87 | 18 | -30 | -83 | 21 | 6.50 | 5.09 |
| Left Lateral Frontal Gyrus | 6/47/9 |  | -36 | -3 | 36 | -36 | -1 | 33 | 6.49 | 5.09 |
| Left Middle Temporal Gyrus | 22 |  | 57 | -33 | 6 | -56 | -32 | 7 | 6.20 | 4.93 |
| Left Cigulate Gyrus | 24 | 59 | -3 | -3 | 33 | -3 | -1 | 30 | 4.62 | 3.98 |
| Right Cingulate Gyrus | 24 |  | 6 | 0 | 33 | 6 | 2 | 30 | 4.22 | 3.71 |
| Right Superior Temporal Gyrus | 38/41/13 | 57 | 60 | 9 | -12 | 59 | 8 | -11 | 4.50 | 3.90 |
| #Left Amygdala |  | 19 | -30 | -6 | -15 | -30 | -6 | -12 | 4.95 | 4.20 |
| #Left Hippocampus |  | 56 | -39 | -27 | -15 | -39 | -27 | -11 | 7.07 | 5.38 |
| #Right Hippocampus |  | 32 | 39 | -12 | -24 | 39 | -13 | -20 | 5.38 | 4.47 |
| #Left Thalamus |  | 199 | -6 | -6 | 9 | -6 | -5 | 9 | 5.73 | 4.67 |
| #Right Thalamus |  | 135 | 15 | -12 | 12 | 15 | -11 | 12 | 5.58 | 4.59 |
| #Left Caudate |  | 91 | -12 | -9 | -15 | -12 | -9 | -12 | 5.48 | 4.53 |
| #Right Caudate |  | 80 | 15 | -6 | 15 | 15 | -5 | 14 | 5.68 | 4.64 |
| #Left Putamen |  | 91 | -12 | -9 | -15 | -12 | -9 | -12 | 5.48 | 4.53 |
| #Right Putamen |  | 80 | 15 | -6 | 15 | 15 | -5 | 14 | 5.68 | 4.64 |
| #Right Cerebellum |  | 41 | 42 | -75 | -21 | 42 | -74 | -14 | 5.27 | 4.40 |

*Note.* BA, Brodmann area. Only clusters (with local maxima coordinates) up to the thresholds of *p* < 0.001 for voxel level (uncorrected), *p* < 0.05 for cluster level (uncorrected), and 50 or more contiguous voxels were reported. # means Small Volume Correction, clusters up to the thresholds of *p* < 0.001 for voxel level (uncorrected) and *p* < 0.05 for cluster level (FWE corrected), 10 or more contiguous voxels were reported.

**Table S3** Regressions between Neural Activities of Metaphor in Encoding and Memory Performances in Testing

| Regressions | Pr | |  | Hit rate | |  | False alarm rate | |  | Accuracy of target theme words | |
| --- | --- | --- | --- | --- | --- | --- | --- | --- | --- | --- | --- |
|  | *β* | *p* |  | *Β* | *p* |  | *Β* | *p* |  | *β* | *p* |
| Left Parahippocampal Gyrus | -0.007 | 0.978 |  | 0.221 | 0.289 |  | 0.232 | 0.219 |  | 0.191 | 0.425 |
| Right Parahippocampal Gyrus | 0.062 | 0.808 |  | 0.138 | 0.408 |  | 0.088 | 0.649 |  | 0.058 | 0.758 |
| Left Hippocampus | 0.001 | 0.998 |  | 0.275 | 0.376 |  | 0.284 | 0.332 |  | 0.333 | 0.344 |
| Right Hippocampus | 0.578 | 0.060 |  | **0.368** | **0.036** |  | -0.202 | 0.377 |  | 0.152 | 0.554 |
| Left Thalamus | 0.186 | 0.264 |  | 0.100 | 0.373 |  | -0.092 | 0.445 |  | 0.045 | 0.753 |
| Right Thalamus | 0.454 | 0.059 |  | 0.318 | 0.068 |  | -0.134 | 0.492 |  | 0.120 | 0.618 |
| Left Amygdala | 0.348 | 0.252 |  | 0.235 | 0.244 |  | -0.115 | 0.655 |  | -0.051 | 0.844 |
| Right Amygdala | 0.307 | 0.283 |  | 0.030 | 0.895 |  | -0.263 | 0.201 |  | -0.056 | 0.824 |
| Left Caudate | 0.425 | 0.260 |  | 0.354 | 0.108 |  | -0.079 | 0.755 |  | 0.381 | 0.132 |
| Right Caudate | **0.782** | **0.004** |  | 0.326 | 0.116 |  | **-0.471** | **0.032** |  | 0.181 | 0.431 |
| Left Putamen | 0.221 | 0.170 |  | 0.131 | 0.313 |  | -0.187 | 0.076 |  | 0.145 | 0.513 |
| Right Putamen | 0.235 | 0.229 |  | 0.116 | 0.302 |  | -0.106 | 0.499 |  | -0.069 | 0.638 |
| Left Cerebellum | **0.469** | **0.017** |  | 0.171 | 0.234 |  | -0.310 | 0.052 |  | 0.094 | 0.616 |
| Right Cerebellum | 0.015 | 0.824 |  | 0.114 | 0.144 |  | 0.101 | 0.071 |  | 0.042 | 0.713 |





**Figure S1** Imaging Results Identified in the Contrast of Metaphor > Literal.
